# Amyand’s Hernia With Sliding Component: A Case Report

**DOI:** 10.7759/cureus.56761

**Published:** 2024-03-23

**Authors:** Imran Ali Khan, Tushar Dahmiwal, Anup Zade, Darshana Tote, Nikhil Thatipalli, Kesav Sudabattula, Srinivasa Reddyy, Shailab Bhadra

**Affiliations:** 1 General Surgery, Jawaharlal Nehru Medical College, Wardha, IND

**Keywords:** inguinal hernia, sliding hernia, mesh, herniorrhaphy, hernioplasty

## Abstract

Amyand's hernia (AH) occurs when the appendix becomes part of an inguinal hernia. Amyand's hernias are typically discovered incidentally during surgery due to their variable clinical manifestations and features, such as caecum and appendix forming the sliding component in the present case. Claudius Amyand operated it for the first time in 1735. Due to the simple presentations that these patients typically exhibit, the diagnosis is extremely challenging. The choice between surgical modalities is influenced by the numerous, logically accepted advantages and disadvantages of management modalities, which are subject to debate. That being said, we believe that, in the absence of sepsis or inflammation, open repair using mesh - as long as a clean operating room and competent surgical skill are available - should be the gold standard approach.

## Introduction

The unusual displacement of an organ or tissue through an opening that is typically responsible for its containment is referred to as a hernia [1]. An incarcerated hernia is a type of non-reducible hernia that contains an organ. If the blood circulation to the incarcerated or edematous colon is impaired, either because of venous or lymphatic blockage, the strangulated hernia may settle in. A catastrophic peritonitis may eventually result from bowel perforation, rupture, and spillage caused by necrosis of the strangulated colon. Amyand's hernia (AH) is the name given to the incarceration of the appendix inside an inguinal hernia (IH). An AH could swell up, get infected, or get ruptured. Additionally, the appendix might be imprisoned and normal [2-4]. The lack of distinct radiological diagnostic features and ambiguous clinical signs and symptoms make definitive preoperative diagnosis a clinical challenge. For instance, an imprisoned appendix or appendicitis that are often misdiagnosed as a strangulated or obstructed hernia [5]. The optimal surgical management strategy is still up for debate, and the diagnosis of AH is still often made during surgery. Doctors have a lot of work ahead of them in learning about AH and its imprisoned appendices because there isn't much information available. Residents and surgeons should possess a thorough understanding of these cases.

## Case presentation

AH with sliding component formed by caecum and appendix was diagnosed in a 65-year-old male patient, a farmer by occupation, who came to the out-patient department with a history of swelling over the sided inguinoscrotal region, which was insidious in onset and gradually progressive in size. The swelling increased on standing/walking and doing strenuous exercise and decreased on lying down. The swelling was not associated with pain. The patient had no history of trauma, fever, breathlessness, chronic cough, constipation, burning micturition, or pain in the abdomen. On inspection, 14x6 cm globular, soft, smooth, partially reducible swelling was found that was doughy in consistency, with expansile cough impulse present. Additionally, there was a right vaginal hydrocele in the scrotum separate from the above swelling. There were no obvious signs of obstruction or strangulation. Right-sided testis was not palpable on examination and showed a positive transillumination test.

**Figure 1:**
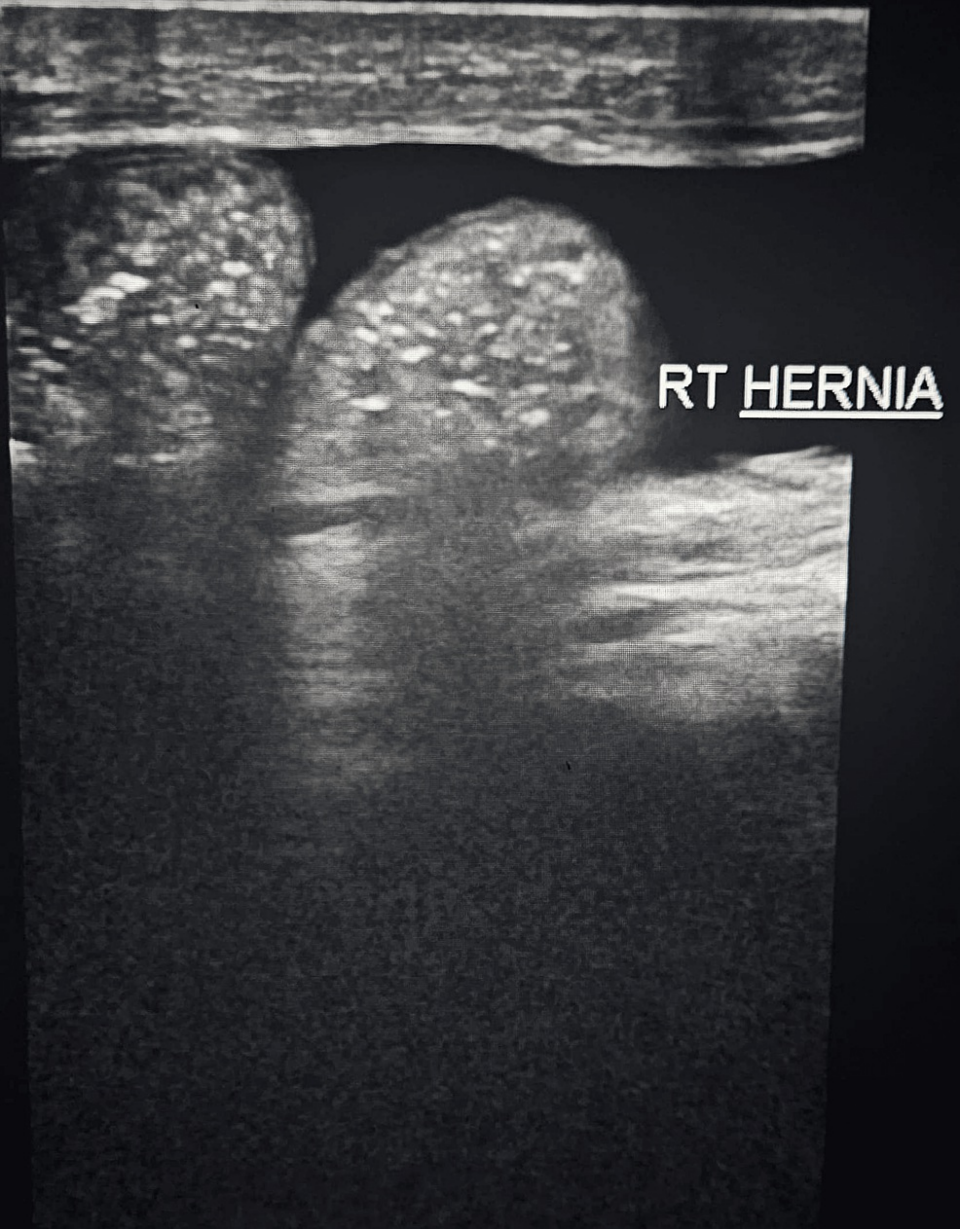
Right-sided inguinoscrotal hernia confirmed on USG scan

**Figure 2:**
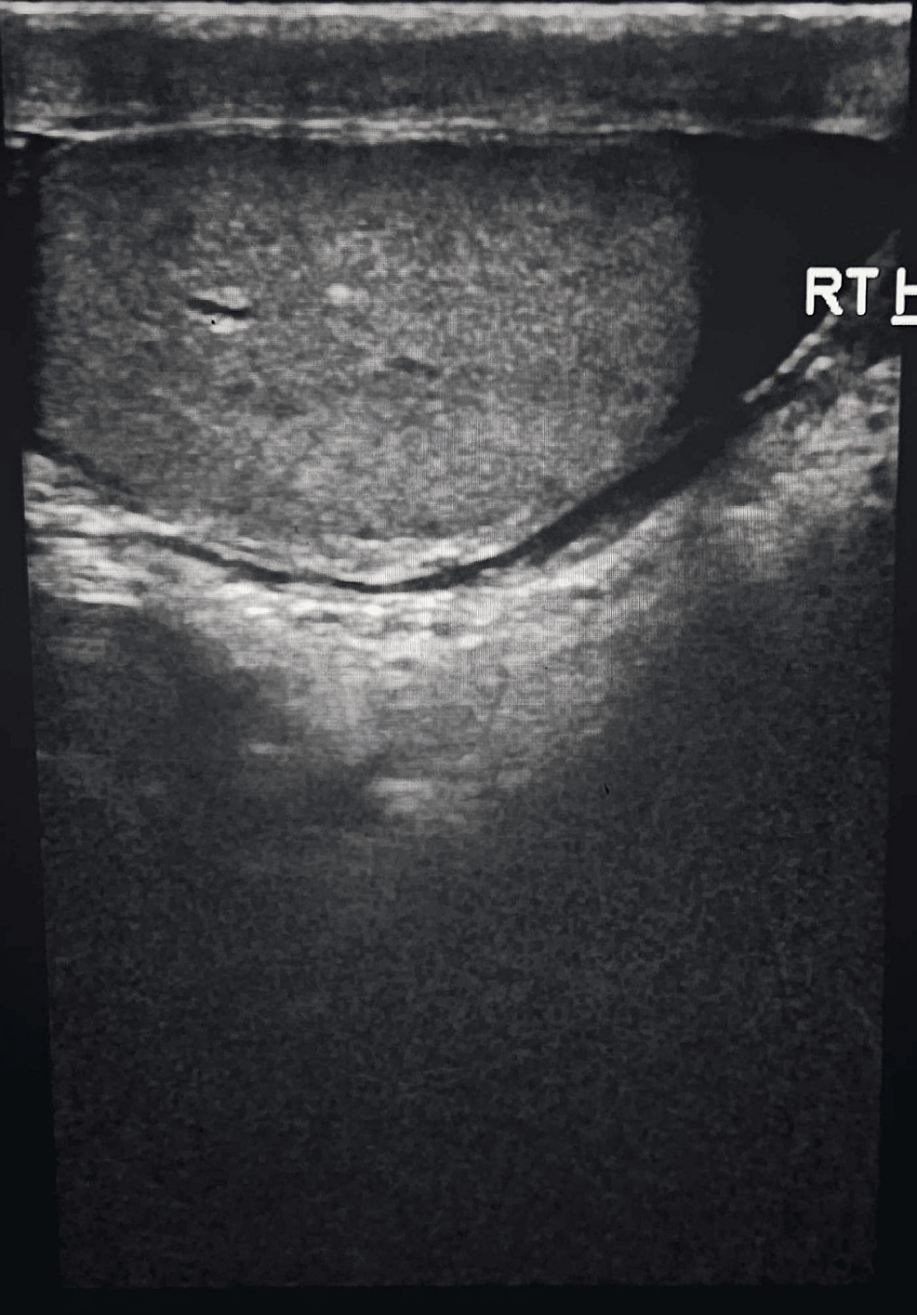
Right-sided hydrocele confirmed on USG scan

Clinical diagnosis of right-sided partially reducible complete inguinoscrotal hernia with bowel and omentum as content (Figure 1), along with right-sided vaginal hydrocele (Figure 2) was made and was confirmed with a USG scan.

**Figure 3:**
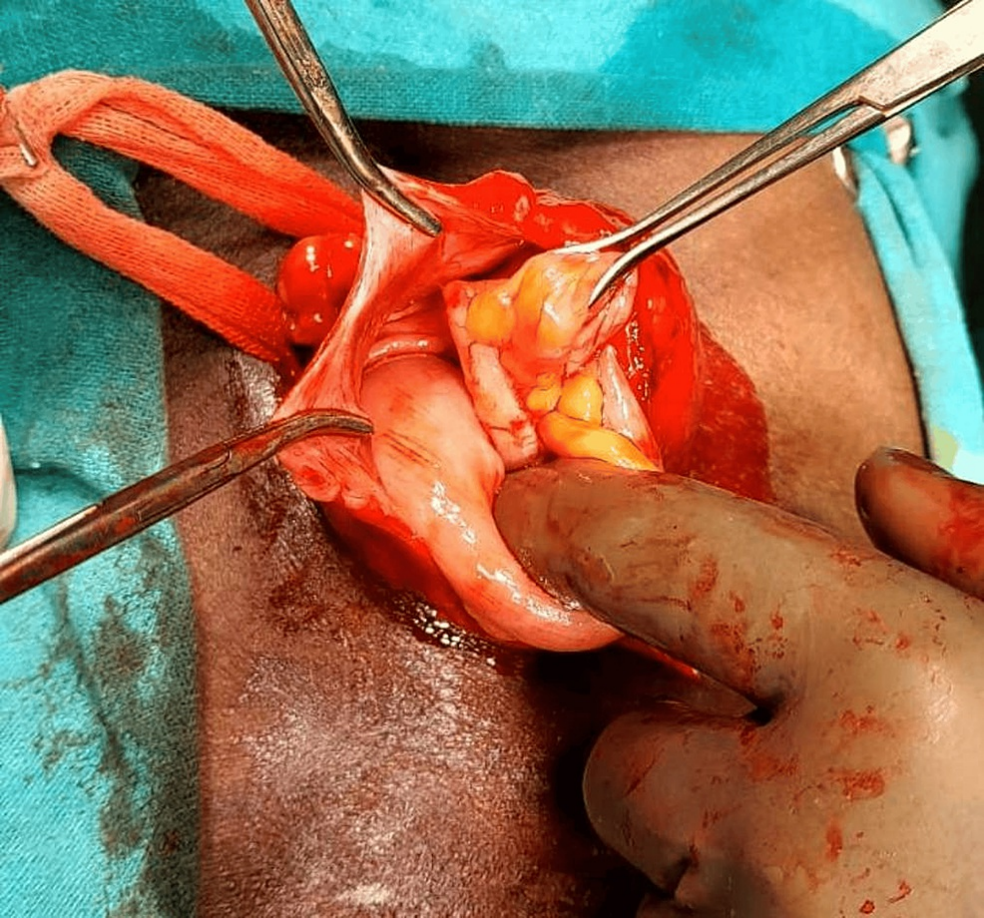
Normal caecum and normal appendix inside hernial sac

All routine blood investigations, including complete blood profile (CBP), coagulation profile, liver function test (LFT), kidney function test (KFT), and chest X-ray, were done and showed no abnormalities. The patient was posted for right-sided inguinal exploration for hernioplasty with Jaboulay's right-sided version of the sac through the same inguinal incision. Open surgery was performed, and the incision was made around 1.6 cm above and parallel to the imaginary line between the anterior superior iliac spine and pubic tubercle. After the diagnosis of AH on opening the sac with the detection of the normal caecum and normal appendix forming the sliding component (Figure 3), the hernia sac was carefully separated from the cord. Transecting the sac into a proximal portion containing AH and a distal portion, the hernia was reduced inside the peritoneal cavity after purse string sutures around the opening of the sac. After the hydrocele was pulled into the inguinal canal, eversion of the sac was performed and the testis was placed back in the right hemiscrotum in its normal anatomical position. Lichtenstein tension-free mesh hernioplasty was performed and the wound was closed in layers after hemostasis. As there was no evidence of appendicitis intra-operatively and the appendix was not found to be inflamed, appendectomy was not indicated and was not done.

The post-operative period was uneventful. Stitches were removed on post-operative day 8 and the patient was discharged. Follow-up examination and radiological imaging showed no sign of recurrence or complications.

## Discussion

By far the most common location for hernias is the inguinal area, which also typically contains the omentum or bowel. The bladder, Meckle’s diverticulum (also called Littre’s hernia), or a section of the intestine circumference (also called Richter’s hernia) are among the unusual contents; however, despite being first documented by Claudius Amyand in 1735, AH is not well known (1).

In 1735, Claudius Amyand became the first person to operate on an IH containing a vermiform appendix and to report the case [3]. AH are rare, with an incidence of 0.19 to 1.7%. The incidence of appendicitis in IH varies from 0.07 to 0.13%. Less frequently, perforated appendix within IH constitutes 0.1% of all appendicitis cases [4-6]. Because of the anatomical position of the appendix, AH commonly manifests on the right side; however, instances of left-sided AH have been made [6].

Losanoff and Basson's classification of Amyand's hernias is depicted in Table 1.

**Table 1:** Losanoff and Basson's classification of Amyand's hernias

Type	Description	Surgical Management
Type 1	Normal appendix in inguinal hernia	Hernia reduction, mesh repair
Type 2	Acute appendicitis in an inguinal hernia, without abdominal sepsis	Appendectomy, primary repair of hernia without mesh
Type 3	Acute appendicitis in an inguinal hernia, with abdominal wall or peritoneal sepsis	Laparotomy, appendectomy, primary repair without mesh
Type 4	Acute appendicitis in an inguinal hernia, with abdominal pathology	Manage as type 1-3, investigate pathology as needed

While various theories exist regarding the pathophysiology of this rare condition, the precise cause is still unknown. Johnson et al. suggested that, in such instances, appendicitis may arise from extraluminal compression rather than an intraluminal obstruction [7].

Particularly in complex cases, a computed tomography (CT) scan is noted for its higher sensitivity and specificity compared to ultrasound. While all these factors contribute to preoperative diagnosis, the ultimate management decision is determined during the intraoperative period.

According to Ivashchuk et al., the best course of treatment should be determined by surgical preference and the existence of appendicitis, and consequently, appendectomy should not limit the utilization of mesh in the repair procedure [2].

AH necessitates appropriate surgical management because the peritoneal spread of sepsis is primarily responsible for the mortality, which ranges from 14 to 30%. Surgery serves both therapeutic and diagnostic purposes, but the best course of action in terms of surgery is still up for debate [8-9].

Although opinions on the best course of action for the management of AH vary, the following questions must be answered: Is an appendectomy necessary? Which strategy ought to be applied? Which kind of repair ought to be done?

AH are rarely diagnosed with imaging studies; instead, the diagnosis is usually made by clinical examination, particularly in cases that are entirely reducible and straightforward. A vermiform appendix within the hernia sac can be found with ultrasound [10].

In this case, as the appendix showed no signs of inflammation, an appendectomy was unnecessary; we performed standard Lichtenstein tension-free hernioplasty with Jaboulay's right-sided sac eversion through the right inguinal incision.

## Conclusions

Amyand's hernias are uncommon and present differently in each individual. The majority of these individuals have simple presentations, which makes the diagnosis extremely challenging. The choice of surgical modality is influenced by the numerous, logically acknowledged advantages and disadvantages of management modalities, which are controversial. However, we believe that in the absence of infection or inflammation, open repair with mesh and additional eversion of the sac of same-sided hydrocele from the same inguinal incision in cases of small to moderate-sized hydrocele - as long as a clean intraoperative environment and competent surgical expertise are present - should be the go-to approach.
